# Variants in genes encoding small GTPases and association with epithelial ovarian cancer susceptibility

**DOI:** 10.1371/journal.pone.0197561

**Published:** 2018-07-06

**Authors:** Madalene Earp, Jonathan P. Tyrer, Stacey J. Winham, Hui-Yi Lin, Ganna Chornokur, Joe Dennis, Katja K. H. Aben, Hoda Anton‐Culver, Natalia Antonenkova, Elisa V. Bandera, Yukie T. Bean, Matthias W. Beckmann, Line Bjorge, Natalia Bogdanova, Louise A. Brinton, Angela Brooks-Wilson, Fiona Bruinsma, Clareann H. Bunker, Ralf Butzow, Ian G. Campbell, Karen Carty, Jenny Chang-Claude, Linda S. Cook, Daniel W Cramer, Julie M. Cunningham, Cezary Cybulski, Agnieszka Dansonka-Mieszkowska, Evelyn Despierre, Jennifer A. Doherty, Thilo Dörk, Andreas du Bois, Matthias Dürst, Douglas F. Easton, Diana M. Eccles, Robert P. Edwards, Arif B. Ekici, Peter A. Fasching, Brooke L. Fridley, Aleksandra Gentry-Maharaj, Graham G. Giles, Rosalind Glasspool, Marc T. Goodman, Jacek Gronwald, Philipp Harter, Alexander Hein, Florian Heitz, Michelle A. T. Hildebrandt, Peter Hillemanns, Claus K. Hogdall, Estrid Høgdall, Satoyo Hosono, Edwin S. Iversen, Anna Jakubowska, Allan Jensen, Bu-Tian Ji, Audrey Y. Jung, Beth Y. Karlan, Melissa Kellar, Lambertus A. Kiemeney, Boon Kiong Lim, Susanne K. Kjaer, Camilla Krakstad, Jolanta Kupryjanczyk, Diether Lambrechts, Sandrina Lambrechts, Nhu D. Le, Shashi Lele, Jenny Lester, Douglas A. Levine, Zheng Li, Dong Liang, Jolanta Lissowska, Karen Lu, Jan Lubinski, Lene Lundvall, Leon F. A. G. Massuger, Keitaro Matsuo, Valerie McGuire, John R. McLaughlin, Iain McNeish, Usha Menon, Roger L. Milne, Francesmary Modugno, Kirsten B. Moysich, Roberta B. Ness, Heli Nevanlinna, Kunle Odunsi, Sara H. Olson, Irene Orlow, Sandra Orsulic, James Paul, Tanja Pejovic, Liisa M. Pelttari, Jenny B. Permuth, Malcolm C. Pike, Elizabeth M. Poole, Barry Rosen, Mary Anne Rossing, Joseph H. Rothstein, Ingo B. Runnebaum, Iwona K. Rzepecka, Eva Schernhammer, Ira Schwaab, Xiao-Ou Shu, Yurii B. Shvetsov, Nadeem Siddiqui, Weiva Sieh, Honglin Song, Melissa C. Southey, Beata Spiewankiewicz, Lara Sucheston-Campbell, Ingvild L. Tangen, Soo-Hwang Teo, Kathryn L. Terry, Pamela J. Thompson, Lotte Thomsen, Shelley S. Tworoger, Anne M. van Altena, Ignace Vergote, Liv Cecilie Vestrheim Thomsen, Robert A. Vierkant, Christine S. Walsh, Shan Wang-Gohrke, Nicolas Wentzensen, Alice S. Whittemore, Kristine G. Wicklund, Lynne R. Wilkens, Yin-Ling Woo, Anna H. Wu, Xifeng Wu, Yong-Bing Xiang, Hannah Yang, Wei Zheng, Argyrios Ziogas, Alice W Lee, Celeste L. Pearce, Andrew Berchuck, Joellen M. Schildkraut, Susan J. Ramus, Alvaro N. A. Monteiro, Steven A. Narod, Thomas A. Sellers, Simon A. Gayther, Linda E. Kelemen, Georgia Chenevix-Trench, Harvey A. Risch, Paul D. P. Pharoah, Ellen L. Goode, Catherine M. Phelan

**Affiliations:** 1 Department of Health Sciences Research, Mayo Clinic, Rochester, MN, United States of America; 2 Department of Oncology, University of Cambridge, Strangeways Research Laboratory, Cambridge, United Kingdom; 3 Department of Biostatistics and Bioinformatics, Moffitt Cancer Center, Tampa, FL, United States of America; 4 School of Public Health, Louisiana State University Health Sciences Center, New Orleans, LA, United States of America; 5 Division of Population Sciences, Department of Cancer Epidemiology, Moffitt Cancer Center, Tampa, FL, United States of America; 6 Netherlands Comprehensive Cancer Organization, Utrecht, The Netherlands; 7 Radboud University Medical Center, Radboud Institute for Health Sciences, Nijmegen, The Netherlands; 8 Genetic Epidemiology Research Institute, UCI Center for Cancer Genetics Research and Prevention, School of Medicine, Department of Epidemiology, University of California Irvine, Irvine, CA, United States of America; 9 Byelorussian Institute for Oncology and Medical Radiology Aleksandrov N.N., Minsk, Belarus; 10 Cancer Prevention and Control, Rutgers Cancer Institute of New Jersey, New Brunswick, NJ, United States of America; 11 Department of Obstetrics and Gynecology, Oregon Health & Science University, Portland, OR, United States of America; 12 Knight Cancer Institute, Oregon Health & Science University, Portland, OR, United States of America; 13 University Breast Center Franconia, Department of Gynecology and Obstetrics, University Hospital Erlangen, Erlangen, Germany; 14 Department of Gynecology and Obstetrics, Haukeland University Hospital, Bergen, Norway; 15 Centre for Cancer Biomarkers, Department of Clinical Medicine, University of Bergen, Bergen, Norway; 16 Gynaecology Research Unit, Hannover Medical School, Hannover, Germany; 17 Division of Cancer Epidemiology and Genetics, National Cancer Institute, Bethesda, MD, United States of America; 18 Canada's Michael Smith Genome Sciences Centre, BC Cancer Agency, Vancouver, BC, Canada; 19 Department of Biomedical Physiology and Kinesiology, Simon Fraser University, Burnaby, BC, Canada; 20 Cancer Epidemiology & Intelligence Division, The Cancer Council Victoria, Melbourne, Australia; 21 Department of Epidemiology, University of Pittsburgh Graduate School of Public Health, Pittsburgh, PA, United States of America; 22 Department of Pathology, Helsinki University Central Hospital, Helsinki, Finland; 23 Department of Obstetrics and Gynecology, University of Helsinki and Helsinki University Central Hospital, Helsinki, Finland; 24 Cancer Genetics Laboratory, Research Division, Peter MacCallum Cancer Centre, Melbourne, Australia; 25 Sir Peter MacCallum Department of Oncology, University of Melbourne, Melbourne, Australia; 26 Department of Pathology, University of Melbourne, Melbourne, Victoria, Australia; 27 CRUK Clinical Trials Unit, The Beatson West of Scotland Cancer Centre, Glasgow, United Kingdom; 28 Department of Gynaecological Oncology, Glasgow Royal Infirmary, Glasgow, United Kingdom; 29 Division of Cancer Epidemiology, German Cancer Research Center (DKFZ), Heidelberg, Germany; 30 University Cancer Center Hamburg, University Medical Center Hamburg-Eppendorf, Hamburg, Germany; 31 Division of Epidemiology and Biostatistics, University of New Mexico, Albuquerque, NM, United States of America; 32 Obstetrics and Gynecology Center, Brigham and Women's Hospital and Harvard Medical School, Boston, MA, United States of America; 33 Department of Laboratory Medicine and Pathology, Mayo Clinic, Rochester, MN, United States of America; 34 International Hereditary Cancer Center, Department of Genetics and Pathology, Pomeranian Medical University, Szczecin, Poland; 35 Department of Pathology, The Maria Sklodowska-Curie Memorial Cancer Center and Institute of Oncology, Warsaw, Poland; 36 Division of Gynecologic Oncology, Department of Obstetrics and Gynecology and Leuven Cancer Institute, University Hospitals Leuven, Leuven, Belgium; 37 Program in Epidemiology, Division of Public Health Sciences, Fred Hutchinson Cancer Research Center, Seattle, WA, United States of America; 38 Huntsman Cancer Institute, University of Utah, Salt Lake City, UT, United States of America; 39 Department of Gynaecology and Gynaecologic Oncology, Dr. Horst Schmidt Kliniken Wiesbaden, Wiesbaden, Germany; 40 Department of Gynaecology and Gynaecologic Oncology, Kliniken Essen-Mitte/ Evang. Huyssens-Stiftung/ Knappschaft GmbH, Essen, Germany; 41 Department of Gynecology, Friedrich Schiller University, Jena, Germany; 42 Centre for Cancer Genetic Epidemiology, Department of Oncology, University of Cambridge, Cambridge, United Kingdom; 43 Centre for Cancer Genetic Epidemiology, Department of Public Health and Primary Care, University of Cambridge, Cambridge, United Kingdom; 44 Wessex Clinical Genetics Service, Princess Anne Hospital, Southampton, United Kingdom; 45 Department of Obstetrics, Gynecology and Reproductive Sciences, University of Pittsburgh School of Medicine, Pittsburgh, PA, United States of America; 46 Institute of Human Genetics, University Hospital Erlangen, Friedrich-Alexander-University Erlangen-Nuremberg, Erlangen, Germany; 47 David Geffen School of Medicine, Department of Medicine Division of Hematology and Oncology, University of California at Los Angeles, CA, United States of America; 48 Department of Cancer Epidemiology, H. Lee Moffitt Cancer Center and Research Institute, Tampa, FL, United States of America; 49 Gynaecological Cancer Research Centre, Department of Women’s Cancer, Institute for Women's Health, University College London, London, United Kingdom; 50 Centre for Epidemiology and Biostatistics, School of Population and Global Health, University of Melbourne, Melbourne, VIC, Australia; 51 Samuel Oschin Comprehensive Cancer Institute, Cedars Sinai Medical Center, Los Angeles, CA, United States of America; 52 Department of Epidemiology, The University of Texas MD Anderson Cancer Center, Houston, TX, United States of America; 53 Department of Gynaecology, Rigshospitalet, University of Copenhagen, Copenhagen, Denmark; 54 Virus, Lifestyle and Genes, Danish Cancer Society Research Center, Copenhagen, Denmark; 55 Molecular Unit, Department of Pathology, Herlev Hospital, University of Copenhagen, Copenhagen, Denmark; 56 Division of Epidemiology and Prevention, Aichi Cancer Center Research Institute, Nagoya, Aichi, Japan; 57 Department of Statistics, Duke University, Durham, NC, United States of America; 58 Women's Cancer Program, Samuel Oschin Comprehensive Cancer Institute, Cedars-Sinai Medical Center, Los Angeles, CA, United States of America; 59 Department of Obstetrics and Gynaecology, University Malaya Medical Centre, University Malaya, Kuala Lumpur, Malaysia; 60 Department of Gynecology, Rigshospitalet, University of Copenhagen, Copenhagen, Denmark; 61 Laboratory for Translational Genetics, Department of Oncology, University of Leuven, Leuven, Belgium; 62 Vesalius Research Center, VIB, University of Leuven, Leuven, Belgium; 63 Division of Gynecologic Oncology; Leuven Cancer Institute, University Hospitals Leuven, KU Leuven, Leuven, Belgium; 64 Cancer Control Research, BC Cancer Agency, Vancouver, BC, Canada; 65 Department of Cancer Prevention and Control, Roswell Park Cancer Institute, Buffalo, NY, United States of America; 66 Gynecology Service, Department of Surgery, Memorial Sloan-Kettering Cancer Center, New York, NY, United States of America; 67 Department of Gynecologic Oncology, The Third Affiliated Hospital of Kunming Medical University (Yunnan Tumor Hospital), Kunming, China; 68 College of Pharmacy and Health Sciences, Texas Southern University, Houston, TX, United States of America; 69 Department of Cancer Epidemiology and Prevention, M. Sklodowska-Curie Memorial Cancer Center & Institute of Oncology, Warsaw, Poland; 70 Department of Gynecologic Oncology, The University of Texas MD Anderson Cancer Center, Houston, TX, United States of America; 71 Radboud University Medical Center, Radboud Institute for Molecular Life Sciences, Nijmegen, The Netherlands; 72 Department of Health Research and Policy, Stanford University School of Medicine, Stanford, CA, United States of America; 73 Public Health Ontario, Toronto, ON, Canada; 74 Womens Cancer Research Program, Magee-Womens Research Institute and University of Pittsburgh Cancer Institute, Pittsburgh, PA, United States of America; 75 The University of Texas School of Public Health, Houston, TX, United States of America; 76 Department of Gynecologic Oncology, Roswell Park Cancer Institute, Buffalo, NY, United States of America; 77 Department of Epidemiology and Biostatistics, Memorial Sloan-Kettering Cancer Center, New York, NY, United States of America; 78 Channing Division of Network Medicine, Brigham and Women's Hospital and Harvard Medical School, Boston, MA, United States of America; 79 Department of Epidemiology, Harvard School of Public Health, Boston, MA, United States of America; 80 Department of Gynecology-Oncology, Princess Margaret Hospital, and Department of Obstetrics and Gynecology, Faculty of Medicine, University of Toronto, Toronto, Ontario, Canada; 81 Department of Population Health Science and Policy, Department of Genetics and Genomic Sciences, Icahn School of Medicine at Mount Sinai, New York, NY, United States of America; 82 Institut für Humangenetik Wiesbaden, Wiesbaden, Germany; 83 Epidemiology Center and Vanderbilt, Ingram Cancer Center, Vanderbilt University School of Medicine, Nashville, TN, United States of America; 84 Cancer Epidemiology Program, University of Hawaii Cancer Center, Honolulu, HI, United States of America; 85 Department of Gynecologic Oncology, Institute of Oncology, Warsaw, Poland; 86 Division of Cancer Etiology and Genetics, National Cancer Institute, Bethesda, MD, United States of America; 87 University Malaya Medical Centre, University Malaya, Kuala Lumpur, Malaysia; 88 Department of Epidemiology, University of Michigan, Ann Arbor, MI, United States of America; 89 Department of Pathology, Rigshospitalet, University of Copenhagen, Copenhagen, Denmark; 90 Department of Obstetrics and Gynecology, Radboud University Medical Center, Nijmegan, The Netherlands; 91 German Cancer Consortium (DKTK) and German Cancer Research Center (DKFZ), Heidelberg, Germany; 92 Department of Health Research and Policy, Department of Biomedical Data Science, Stanford University School of Medicine, Stanford, CA, United States of America; 93 Cancer Research Malaysia, Subang Jaya Selangor, Malaysia; 94 Department of Preventive Medicine, Keck School of Medicine, University of Southern California, Los Angeles, CA, United States of America; 95 Department of Epidemiology, Shanghai Cancer Institute, Shanghai, China; 96 Vanderbilt Epidemiology Center, Vanderbilt University School of Medicine, Nashville, TN, United States of America; 97 Department of Health Science, California State University, Fullerton, Fullerton, CA, United States of America; 98 Department of Obstetrics and Gynecology, Duke University Medical Center, Durham, NC, United States of America; 99 Department of Public Health Sciences, The University of Virginia, Charlottesville, VA, United States of America; 100 School of Women's and Children's Health, University of New South Wales, Sydney, Australia; 101 The Garvan Institute, Sydney, New South Wales, Australia; 102 Women's College Research Institute, University of Toronto, Toronto, Ontario, Canada; 103 Center for Cancer Prevention and Translational Genomics, Samuel Oschin Comprehensive Cancer Institute, Cedars-Sinai Medical Center, Los Angeles, CA, United States of America; 104 Department of Public Health Sciences, Medical University of South Carolina and Hollings Cancer Center, Charleston, SC, United States of America; 105 Department of Genetics, QIMR Berghofer Medical Research Institute, Brisbane, Australia; 106 Department of Chronic Disease Epidemiology, Yale School of Public Health, New Haven, CT, United States of America; 107 Department of Public Health and Primary Care, University of Cambridge, Strangeways Research Laboratory, Worts Causeway, Cambridge, United Kingdom; University of Newcastle, AUSTRALIA

## Abstract

Epithelial ovarian cancer (EOC) is the fifth leading cause of cancer mortality in American women. Normal ovarian physiology is intricately connected to small GTP binding proteins of the Ras superfamily (Ras, Rho, Rab, Arf, and Ran) which govern processes such as signal transduction, cell proliferation, cell motility, and vesicle transport. We hypothesized that common germline variation in genes encoding small GTPases is associated with EOC risk. We investigated 322 variants in 88 small GTPase genes in germline DNA of 18,736 EOC patients and 26,138 controls of European ancestry using a custom genotype array and logistic regression fitting log-additive models. Functional annotation was used to identify biofeatures and expression quantitative trait loci that intersect with risk variants. One variant, *ARHGEF10L* (Rho guanine nucleotide exchange factor 10 like) rs2256787, was associated with increased endometrioid EOC risk (OR = 1.33, p = 4.46 x 10^−6^). Other variants of interest included another in *ARHGEF10L*, rs10788679, which was associated with invasive serous EOC risk (OR = 1.07, p = 0.00026) and two variants in *AKAP6* (A-kinase anchoring protein 6) which were associated with risk of invasive EOC (rs1955513, OR = 0.90, p = 0.00033; rs927062, OR = 0.94, p = 0.00059). Functional annotation revealed that the two *ARHGEF10L* variants were located in super-enhancer regions and that *AKAP6* rs927062 was associated with expression of GTPase gene *ARHGAP5* (Rho GTPase activating protein 5). Inherited variants in *ARHGEF10L* and *AKAP6*, with potential transcriptional regulatory function and association with EOC risk, warrant investigation in independent EOC study populations.

## Introduction

In 2017, in the United States, more than 21,000 women were expected to be diagnosed with epithelial ovarian cancer (EOC), and more than 14,000 women were predicted to die from the disease.[1] EOC is heterogeneous and therefore classified into major histological subtypes of invasive disease—serous, endometrioid, clear cell, and mucinous–and two histological subtypes of borderline disease–serous and mucinous. These histological subtypes have differences in genetic and epidemiologic risk factors, molecular events during oncogenesis, response to chemotherapy, and prognosis.[2]

Approximately 20% of the familial component of EOC risk is attributable to high-to-intermediate risk gene mutations.[3] In European populations, genome-wide association studies (GWAS) have identified more than 30 EOC susceptibility alleles, as reviewed previously.[4] Known common genetic variants explain 3.9% of the inherited component of EOC risk, and additional susceptibility loci are likely to exist, particularly for the less common, non-serous histological subtypes.

Normal ovarian physiology is intricately connected to tightly regulated small GTP binding proteins of the Ras superfamily (Ras, Rho, Rab, Ral, Arf, and Ran) which regulate key cellular processes such as signal transduction, cell proliferation, cell motility, and vesicle transport.[5] These proteins function in a highly coordinated manner through signaling networks and feedback loops within and among the small GTPase subfamilies.[6] The Rab and Ral GTPases are thought to function in membrane trafficking in exocyst assembly and vesicle-tethering processes;[7, 8] Rho-related proteins function to integrate extracellular signals with specific targets regulating cell morphology, cell aggregation, tissue polarity, cell motility and cytokinesis.[5] Ras family genes cycle between their inactive GDP forms in the cytoplasm and the active GTP-bound forms on the plasma membrane and are associated with signaling pathways contributing to normal and aberrant cell growth.[9]

As regulation of the RAS signal transduction pathway involves a highly complex, highly polymorphic machinery of genes, we conducted a large-scale candidate pathway association study, hypothesizing that variation in small GTPase genes is associated with EOC risk.

## Materials and methods

### Variant selection

RAS pathway genes were selected based on the Cancer Genome Anatomy Project and review of the published literature (www.pubmed.gov). Within 115 candidate genes, 6103 single nucleotide polymorphism (SNPs) were interrogated in early GWAS analysis of 7931 EOC patients and 9206 controls;[10] 339 SNPs in 88 of these genes showed nominal evidence of association with risk of EOC or of serous EOC (p<0.05 using all participants or North American participants only)[10] and were targeted in the present analysis (S1 Table).

### Study participants and genotyping

We studied 18,736 EOC patients (10,316 of serous histology) and 26,138 controls who participated in Ovarian Cancer Association Consortium studies; all participants were of European ancestry.[11] This included participants from the GWAS which was used for variant selection (described above)[10] and an additional 10,243 patients and 16,932 controls. Genotyping used a custom Illumina Infinium array. [11] SNPs were excluded according to the following criteria: no genotype call; monomorphism; call rate less than 95% and minor allele frequency > 0.05 or call rate less than 99% with minor allele frequency < 0.05; evidence of deviation of genotype frequencies from Hardy-Weinberg equilibrium (p < 10^−7^); greater than 2% discordance in duplicate pairs. Overall, 322 small GTPase gene SNPs were genotyped and passed QC; numbers of participants with data for each SNP vary, as some DNA samples failed QC for particular SNPs. This study was reviewed and approved by the Mayo Clinic Institutional Review Board as protocol 1367–05.

### Genetic association

We followed STREGA guidelines for genetic association studies.[12] Unconditional logistic regression treating the number of minor alleles carried as an ordinal variable (log-additive model) was used to evaluate the association between each SNP and EOC risk adjusted for age, study site, and principal components to account for residual differences in European ancestry. Six series of analyses were conducted considering the following groups: all invasive EOC combined, each of the four main invasive histological subtypes (serous, endometrioid, clear cell and mucinous), and all borderline tumors combined. No corrections were made for multiple testing.

### Functional annotation

For SNPs of interest, dbSUPER [13] and Haploreg v4.1[14] were used to evaluate publicly available data for variant overlap with human super-enhancers,[15] known expression quantitative trait loci (eQTL), GWAS hits, and other regulatory marks. In addition, we assessed correlations between germline genotype with tumor expression levels (eQTL analysis) using 312 Mayo Clinic patients (226 serous, 54 endometrioid, 22 clear cell, 5 mucinous, and 5 of other histological subtypes). Expression data were obtained using fresh frozen tumor RNA and Agilent whole human genome 4×44 expression arrays and were analyzed in the form of log ratios of signals from individual tumors compared to signals from a reference mix of 106 tumor samples[16, 17] versus signals from a reference mix of 106 tumor samples[16, 17]. Expression levels for minor allele carriers versus non-carriers were compared using the Wilcoxon rank sum statistic.

## Results and discussion

Demographic and clinical characteristics of the study sample (18,736 EOC patients and 26,138 controls) have been described previously.[11] In brief, compared to controls, patients were older, attained menarche at older ages, and had higher body mass index. As expected, most tumors (57.6%) were of serous histology with 14.2% endometrioid, 7.1% clear cell, 6.5% mucinous, and 14.6% other/unknown.

From among 322 SNPs in 88 RAS pathway small *GTPase* genes, we observed that 99 SNPs in 43 genes were nominally associated with EOC risk (p<0.05) (S2 Table). These associations were from six separate analyses that evaluated all patients with invasive disease, patients with one of the four main invasive histological subtypes, serous [n = 8,372], endometrioid [n = 2,068], clear cell [n = 1,025] and mucinous [n = 943], as well as patients with borderline tumors.

**Fig 1:**
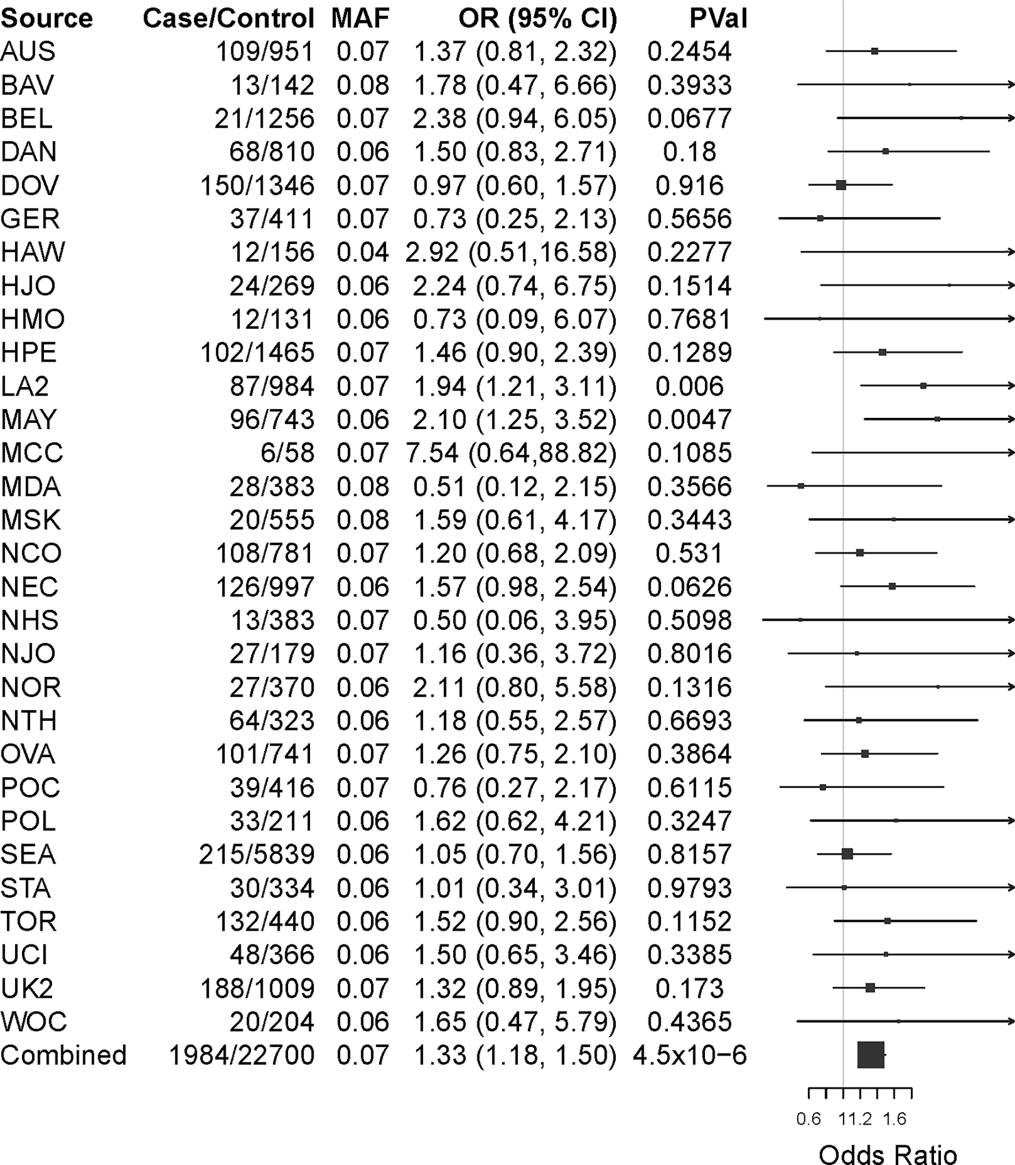
Association of rs2256787 in the *ARHGEF10L* gene with invasive endometrioid EOC risk by study site and combined. Squares represent the estimated per-allele odds ratio (OR) and are proportional to sample size for each study; lines indicate its 95% confidence interval (CI); source indicates contributing study;[11] MAF, control minor allele frequency; PVal, per-allele p-value adjusted for age, site, and principal components to account for residual differences in European ancestry.

In *ARHGEF10L*, which encodes the Rho guanine nucleotide exchange factor 10-like protein, SNP rs2256787 was associated with invasive endometrioid EOC risk (OR = 1.33, 95% CI: 1.18–1.50, p = 4.5x10^-6^) (Table 1). (Fig 1) shows the ORs and 95% CIs associated with the G allele at this SNP overall and by contributing study.

**Table 1:** Association of variants in small GTPase genes with epithelial ovarian cancer risk (p-value<10^−4^) and functional annotation.

					Genetic Association			Functional Annotation				
Gene	SNP	Chr:Position	Alleles	MAF	Histology	OR (95% CI)	P-value	Conserved site	eQTL	Tissues with enhancer histone mark	Tissues with DNAse site	In super-enhancer
***ARHGEF10L***	rs2256787	1:17,765,403	A/C	0.07	Endometrioid	1.33 (1.18–1.50)	4.5 x 10^−6^	No	No	ESC, ESDR, IPSC, FAT, STRM, BRST, BRN, SKIN, VAS, LIV, GI, HRT, MUS, LNG, OVRY, PANC	None	Yes
	rs10788679	1:17,789,549	A/G	0.42	Serous	1.07 (1.03–1.11)	2.6 x 10^−4^	No	No	None	None	Yes
***AKAP6***	rs1955513	14:32,245,693	C/A	0.07	All invasive	0.90 (0.85–0.95)	3.3 x 10^−4^	Yes	No	FAT, SKIN, VAS, BRN, MUS, GI, BLD	SKIN,MUS,MUS,THYM,BLD	No
	rs927062	14:32,164,800	G/A	0.21	All invasive	0.94 (0.90–0.97)	5.9 x 10^−4^	No	Yes, ARHGAP5	None	GI	No

SNP, single nucleotide polymorphism; alleles show minor/major; MAF, minor allele frequency; OR, odds ratio; CI, confidence interval; eQTL, expression quantitative locus with p<0.05 in EOC tumors; histone marks and DNAse I hypersensitive sites from HaploReg v 4.1 indicating tissue types as defined therein; super enhancer information based on the human super-enhancer database available at http://bioinfo.au.tsinghua.edu.cn/dbsuper/index.php; none of these SNPs had previous GWAS associations with any phenotype based on the EBI GWAS catalog or resided within promoter histone marks; all SNPs are intronic to the gene indicated.

Three other variants were associated at p-value<10^−4^ (Table 1, S1, S2 and S3 Figs). rs10788679 in an intron of *ARHGEF10L* was associated with risk of invasive serous EOC (OR = 1.07, 95% CI: 1.03–1.11, p = 2.6x10^-4;^); *ARHGEF10L* SNPs rs2256787 and rs10788679 are independent (r^2^ = 0.02, 1000 Genomes Project EUR). In addition, rs1955513 was most strongly associated with all invasive EOC risk (OR = 0.90, 95% CI: 0.85–0.95, p = 3.3x10^-4^). This variant lies in an intron of A-kinase (PRKA) anchor protein 6 (*AKAP6*). Another variant in *AKAP6*, intronic SNP rs927062, was also associated with all invasive EOC risk (p = 5.9x10^-4^); *AKAP6*SNPs rs1955513 and rs927062 are in modest linkage disequilibrium (r^2^ = 0.15, 1000 Genomes Project EUR).

We investigated whether the four variants of interest, rs2256787, rs10788679, rs1955513, rs927062, which are all intronic, alter expression of their proximal GTPases, or coincide with regulatory marks that may affect expression (Table 1). In publicly available databases,[13, 14] the *ARHGEF10L* SNPs rs2256787and rs10788679 coincide with a human ovary super-enhancer, a region of the genome with unusually strong enrichment for the binding of transcriptional coactivators in this tissue. As *ARHGEF10L* rs2256787 associated with endometrioid EOC risk, we were particularly interested in eQTLs in the 54 endometrioid patients; however, there was no evidence of association between rs2256787 genotype and *ARHGEF10L* expression in endometrioid EOC tumors or other tumor subtypes. In 312 invasive EOC tumors, the G allele of *AKAP6* rs927062 correlated with reduced expression of Rho GTPase activating protein 5 (ARHGAP5), a GTPase ~150kb upstream of *AKAP6* (β = -0.22, 95% CI: -0.41 to -0.03, p = 6.6x10^-3^). Other unstudied variants may also be associated with expression of *ARHGAP5* (or may be more strongly associated than rs927062), thus future genome-wide or pathway-based analysis of GTPase SNP-expression relationships are of great interest. In other histology-specific eQTL analyses, none of the four variants tested were associated with EOC tumor mRNA expression.

## Conclusion

We investigated 322 SNPs in 88 genes encoding small GTP binding proteins of the Ras superfamily (Ras, Rho, Rab, Ral, Arf, and Ran) in germline DNA of over 17,000 EOC patients and 26,000 controls. The 88 genes were derived from G protein (guanine nucleotide-binding proteins) signaling, Ras-GTPases, regulation of Rho GTPase protein signal transduction and activation of Rac GTPase activity. [18] Ras-GTPases are activated at the plasma membrane by guanine nucleotide exchange factors (GEF) such as: son of sevenless homologs 1 and 2 (Drosophila) (SOS-1 and SOS-2); Ras protein-specific guanine nucleotide-releasing factor 1 (GRF1); Rap guanine nucleotide exchange factor 1 (GRF2); and RasGEF domain family, members 1A, 1B and 1C (RasGRF). They are inactivated by GTPase activating proteins (GAP) which include RAS p21 protein activator (GTPase activating protein) 1 (p120RasGAP). GEF factors are recruited to the plasma membrane by scaffold and adaptor complexes such as SHC/Grb2 that associate with activated tyrosine kinase receptors (TKR).[19] These factors exchange GTP for GDP on the Ras protein. The resulting GTP-Ras protein activates various downstream effectors such as MAP-kinase Raf-1 which activates the MEK/ERK gene regulation cascade, a primary cell growth and anti-apoptosis pathway.[6] Ras-GTPases family members regulate the action of other GTPase pathways involving Rap, Ral, Rac and Rho Ras-GTPase. Ras-GTPases also regulate phosphoinositide 3-kinase (PI3K) and phospholipase C (PLC) activities.[5] Several of these genes are mutated in ovarian tumors.[20]

Overall, analysis at only one SNP yielded a p-value < 10^−5^: rs2256787 in *ARHGEF10L* which was associated with 33% increased endometrioid EOC risk. Of note, the experiment-wide error rate for this SNP, accounting for the initial overall set of 6103 candidate SNPs equals 0.027 (Bonferroni-corrected p-value 4.5 x 10^−6^ x 6103); additionally accounting for six case groups analyzed, this value increases to 0.16 (Bonferroni-corrected p-value 4.5 x 10^−6^ x 6103 x 6). However, as SNPs, as well as case groups, are not independent, simulation studies are necessary to derive an empirical p-value. Another *ARHGEF10L* SNP, rs10788679, in showed the smallest p-value in analysis of serous EOC and was the second-most strongly associated SNP in all analyses. ARHGEF10L is a member of the RhoGEF family GEFs that activate Rho GTPases.[21] The Rho branch of the Ras super family encompasses 20 genes in humans, of which Rho, Rac and Cdc42 are the best characterized. Rho GTPases regulate the actin cytoskeleton and control changes in cell morphology and cell motility triggered by extracellular stimuli. Rho GTPases are regulated by GDP/GTP exchange factors and GAPs. Members of this subfamily are activated by specific GEFs and are involved in signal transduction. SNPs in this gene are also associated with obesity[22] and cutaneous basal cell carcinoma.[23]

The SNP most associated with risk of invasive EOC was rs1955513 in the *AKAP6* gene. This gene is involved in overall G protein signaling. SNPs in this gene are also associated with neurologic functioning [24] and anorexia.[25] Functionally, rs927062 in *AKAP6* was associated with expression of the Rho GTPase activating protein 5, *ARHGAP5*, also known as p190 RhoGAP, which negatively regulates RHO GTPases. The p190 RhoGAP gene contains a carboxy-terminal domain that functions as a GAP for the Rho family GTPases. In addition to its RhoGAP domain, p190 contains an amino-terminal domain that contains sequence motifs found in all known GTPases.

In conclusion, our study identified potentially functional genetic variants in small GTPase genes that may have roles in EOC susceptibility. To interpret these associations, we suggest consideration of effect sizes and directionality in the context of the sets of histotype-specific analyses conducted; whether a more conservative or liberal statistical significance threshold is applied, the small set of variants highlighted for detailed functional follow-up remain the same. A limitation of this work is that nearby imputed variants were not examined and thus other ungenotyped variants may be driving the reported associations. Nonetheless, four variants in two genes show promising associations that have not been reported previously but point to known pathways that are mutated in ovarian tumors. The results of our investigation suggest that further assessment of this important pathway is warranted in additional collections of densely genotyped EOC patients and controls.

## Supporting information

S1 Fig Association of rs10788679 in the *ARHGEF10L* gene with invasive serous EOC risk by study site and combined. Squares represent the estimated per-allele odds ratio (OR) and are proportional to sample size for each study; lines indicate its 95% confidence interval (CI); Source indicates contributing study [11]; MAF, control minor allele frequency; PVal, per-allele p-value adjusted for age, site, and residual European principal components.
(TIFF)

S2 Fig Association of rs1955513 in the *AKAP6* gene with invasive EOC risk by study site and combined. Squares represent the estimated per-allele odds ratio (OR) and are proportional to sample size for each study; lines indicate its 95% confidence interval (CI); Source indicates contributing study [11]; MAF, control minor allele frequency; PVal, per-allele p-value adjusted for age, site, and residual European principal components.
(TIFF)

S3 Fig Association of rs927062 in the *AKAP6* gene with invasive EOC risk by study site and combined. Squares represent the estimated per-allele odds ratio (OR) and are proportional to sample size for each study; lines indicate its 95% confidence interval (CI); Source indicates contributing study [11]; MAF, control minor allele frequency; PVal, per-allele p-value adjusted for age, site, and residual European principal components.
(TIFF)

S1 Table Results from prior published EOC GWAS results on the targeted 339 SNPs in 88 RAS pathway genes. More details are available upon request.
(XLS)

S2 Table Results from EOC genetic association analysis on 99 SNPs in RAS pathway genes with nominal p-value <0.05 in analysis of all invasive patients, patients with invasive serous, endometrioid, clear cell, or mucinous subtypes, and patients with borderline tumors versus controls. More details are available upon request.
(XLSX)
