# Supplementary figures and images for: Variants in genes encoding small GTPases and association with epithelial ovarian cancer susceptibility
Source: PLoS One. 2018 Jul 6;13(7):e0197561. doi: 10.1371/journal.pone.0197561 (PMC6034790; doi:10.1371/journal.pone.0197561)

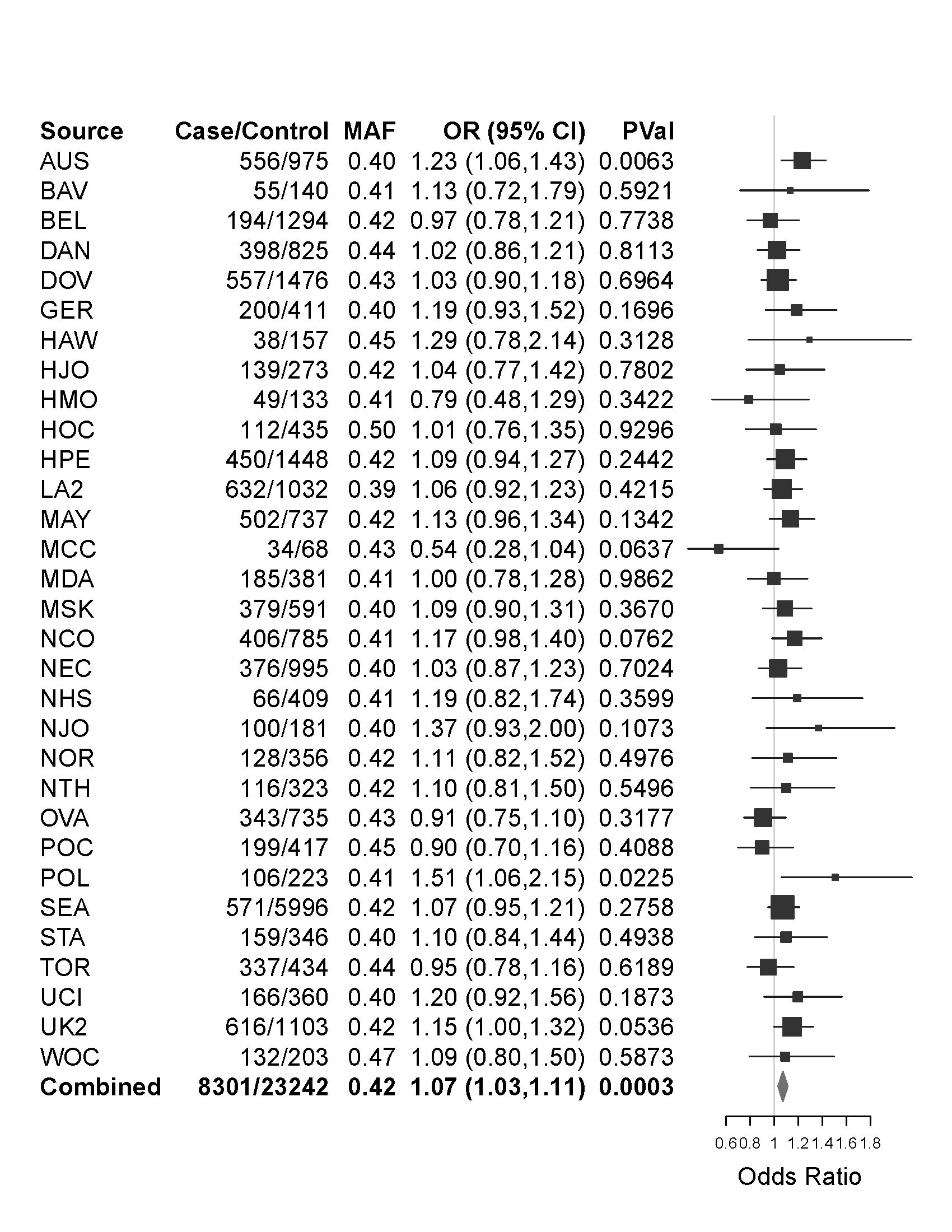

Supplement: S1 Fig — Squares represent the estimated per-allele odds ratio (OR) and are proportional to sample size for each study; lines indicate its 95% confidence interval (CI); Source indicates contributing study [11]; MAF, control minor allele frequency; PVal, per-allele p-value adjusted for age, site, and residual European principal components. (TIFF) [file pone.0197561.s001.tiff]

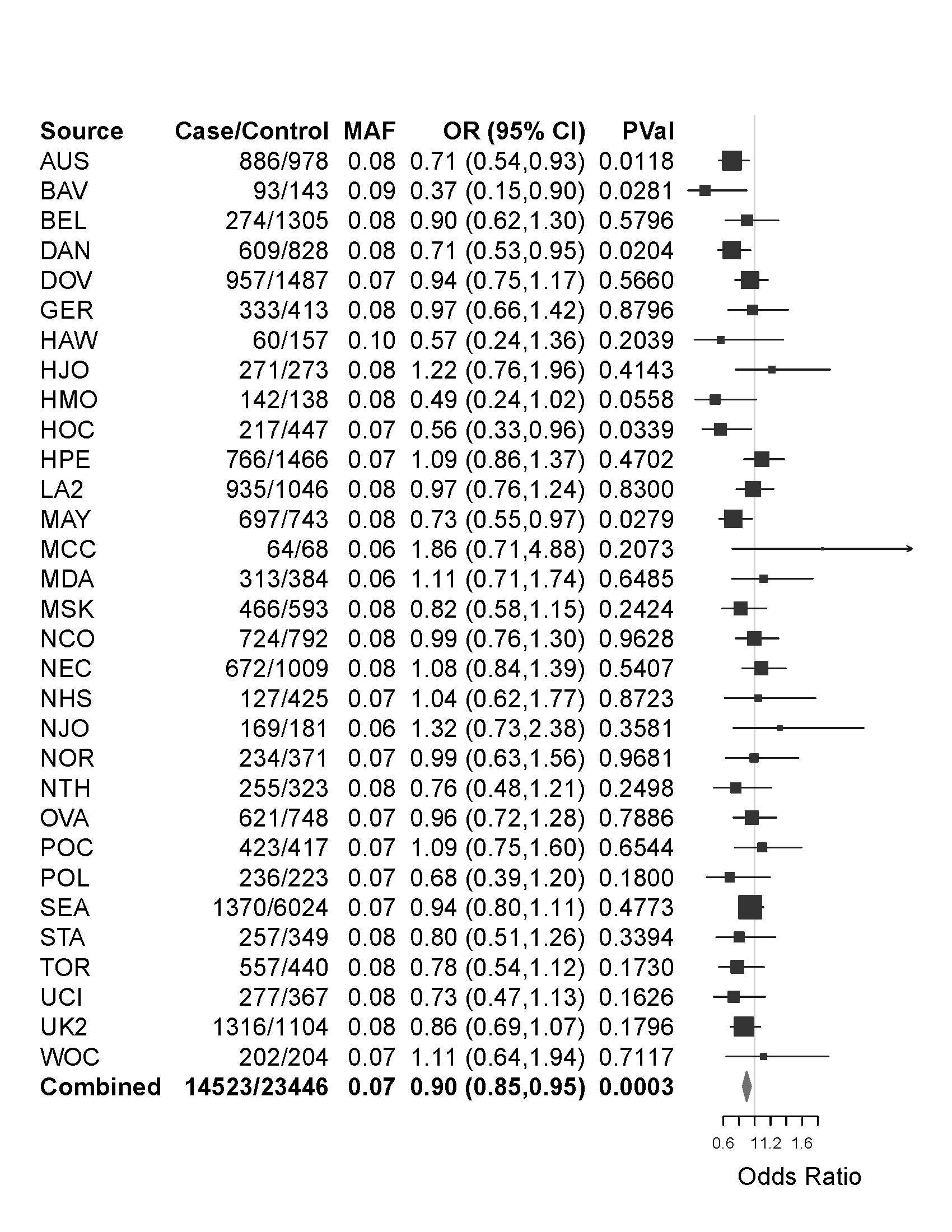

Supplement: S2 Fig — Squares represent the estimated per-allele odds ratio (OR) and are proportional to sample size for each study; lines indicate its 95% confidence interval (CI); Source indicates contributing study [11]; MAF, control minor allele frequency; PVal, per-allele p-value adjusted for age, site, and residual European principal components. (TIFF) [file pone.0197561.s002.tiff]

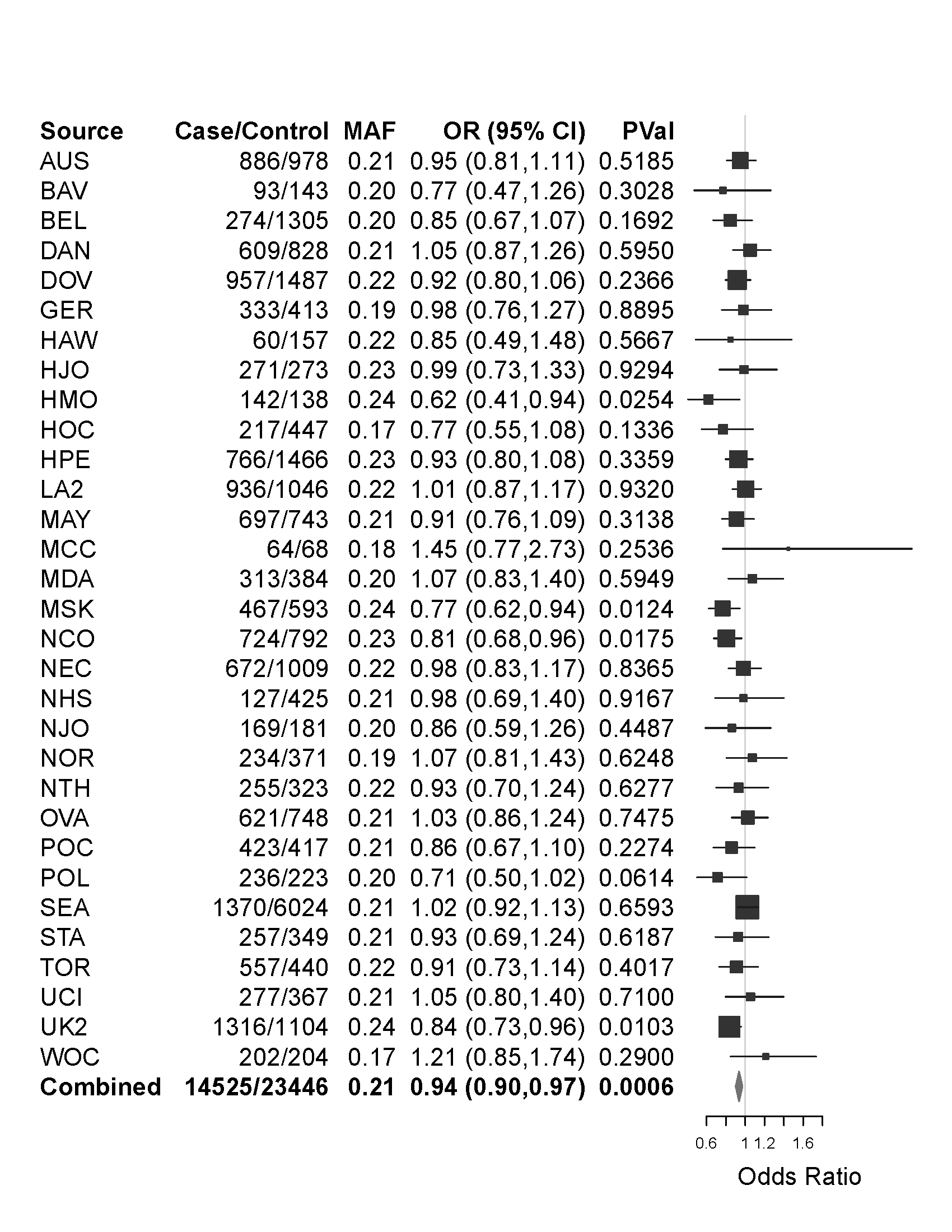

Supplement: S3 Fig — Squares represent the estimated per-allele odds ratio (OR) and are proportional to sample size for each study; lines indicate its 95% confidence interval (CI); Source indicates contributing study [11]; MAF, control minor allele frequency; PVal, per-allele p-value adjusted for age, site, and residual European principal components. (TIFF) [file pone.0197561.s003.tiff]
